# Influence of a Double-Lumen Extension Tube on Drug Delivery: Examples of Isosorbide Dinitrate and Diazepam

**DOI:** 10.1371/journal.pone.0154917

**Published:** 2016-05-06

**Authors:** Aurélie Maiguy-Foinard, Nicolas Blanchemain, Christine Barthélémy, Bertrand Décaudin, Pascal Odou

**Affiliations:** 1 Univ. Lille, EA 7365 - GRITA - Groupe de Recherche sur les formes Injectables et les Technologies Associées, F-59000 Lille, France; 2 CHU Lille, Institut de Pharmacie, F-59000 Lille, France; 3 Univ. Lille, F-59000 Lille, France; 4 Inserm, U1008 - Controlled Drug Delivery Systems and Biomaterials, F-59000 Lille, France; Laurentian, CANADA

## Abstract

**Purpose:**

Plastic materials such as polyurethane (PUR), polyethylene (PE), polypropylene (PP) and polyvinyl chloride (PVC) are widely used in double-lumen extension tubing. The purposes of our study were to 1) compare in vitro drug delivery through the double extension tubes available on the market 2) assess the plastic properties of PUR in infusion devices and their impact on drug delivery.

**Methods:**

The study compared eight double-lumen extension tubes in PUR, co-extruded (PE/PVC) plastic and plasticised PVC from different manufacturers. Isosorbide dinitrate and diazepam were used as model compounds to evaluate their sorption on the internal surface of the infusion device. Control experiments were performed using norepinephrine known not to absorb to plastics. Drug concentrations delivered at the egress of extension tubes were determined over time by an analytical spectrophotometric UV-Vis method. The main characteristics of plastics were also determined.

**Results:**

Significant differences in the sorption phenomenon were observed among the eight double-lumen extension tubes and between pairs of extension tubes. Mean concentrations of isosorbide dinitrate delivered at the egress of double-lumen extension tubes after a 150-minute infusion (mean values ± standard deviation in percentage of the initial concentrations in the prepared syringes) ranged between 80.53 ± 1.66 (one of the PUR tubes) and 92.84 ± 2.73 (PE/PVC tube). The same parameters measured during diazepam infusion ranged between 48.58 ± 2.88 (one of the PUR tubes) and 85.06 ± 3.94 (PE/PVC tube). The double-lumen extension tubes in PUR were either thermosetting (resin) or thermoplastic according to reference.

**Conclusions:**

Clinicians must be aware of potential drug interactions with extension tube materials and so must consider their nature as well as the sterilisation method used before selecting an infusion device.

## Introduction

Patients in intensive care units receive many drugs simultaneously, resulting in the need for multi-lumen extension tubes. The main criterion of choice for these devices appears to be their dead volume, defined as the volume between the meeting point of the drugs simultaneously infused and the egress of the device. [1–3] This parameter is related to the delay to reach the steady state in drug delivery after a change in drug flow during multi-infusion therapy. [4] However, other characteristics of the device affect drug delivery and have to be taken into account.

Plastic materials such as polyurethane (PUR), polyethylene (PE), polypropylene (PP) and polyvinyl chloride (PVC) are widely used in various medical devices. Plasticised PVC is the most common material for infusion devices. However, its composition has changed recently with doubts concerning the use of di-(2-ethylhexyl) phthalate (DEHP) and other hazardous phthalate plasticisers. [5–7] DEHP has been replaced by alternatives and in some cases, plasticised PVC has been replaced by other materials. The evolution in infusion device materials may affect drug-device interaction and so drug delivery, especially with possible drug adsorption on the inner plastic surface of the container. This phenomenon leads indeed to drug loss and results in reduced drug delivery to the patient. [8–10] The nature of the plastic material is an important parameter because it determines the type and the amount of drug binding. Plasticised PVC has high potential interaction with drugs, whereas PE or PP materials are subject to less. [11–13] Other criteria have to be taken into account, especially the mechanical properties of the materials, their ease of assembly and their manufacturing costs.

Different techniques are used to sterilise infusion devices (ethylene oxide (EtO) gas or gamma irradiation (GI)) and these may have harmful effects on medical grade polymers such as extensive material degradation and plastic deformation/modification affecting drug-device interaction. [14–16] Sterilisation techniques can either act physically or chemically leading to alterations in the structure of macromolecules, which can result in chain scission, oxidation, crosslinking, melting or hydrolysis. [17–19] The susceptibility of plastics to the process of high energy radiosterilisation has been shown to be closely dependent on the chemical structure and purity of the material. [19] Studies have shown that PUR provides good irradiation resistance thanks to its high degree of crosslinking. [20]

As many multi-lumen extension tubes with different materials and sterilisation processes are available, the purposes of our study were, firstly, to measure the concentration of two drugs after passing through devices available on the market and, secondly, to determine the nature of the PUR used in the infusion devices and its impact on drug delivery.

## Methods

**Fig 1:**
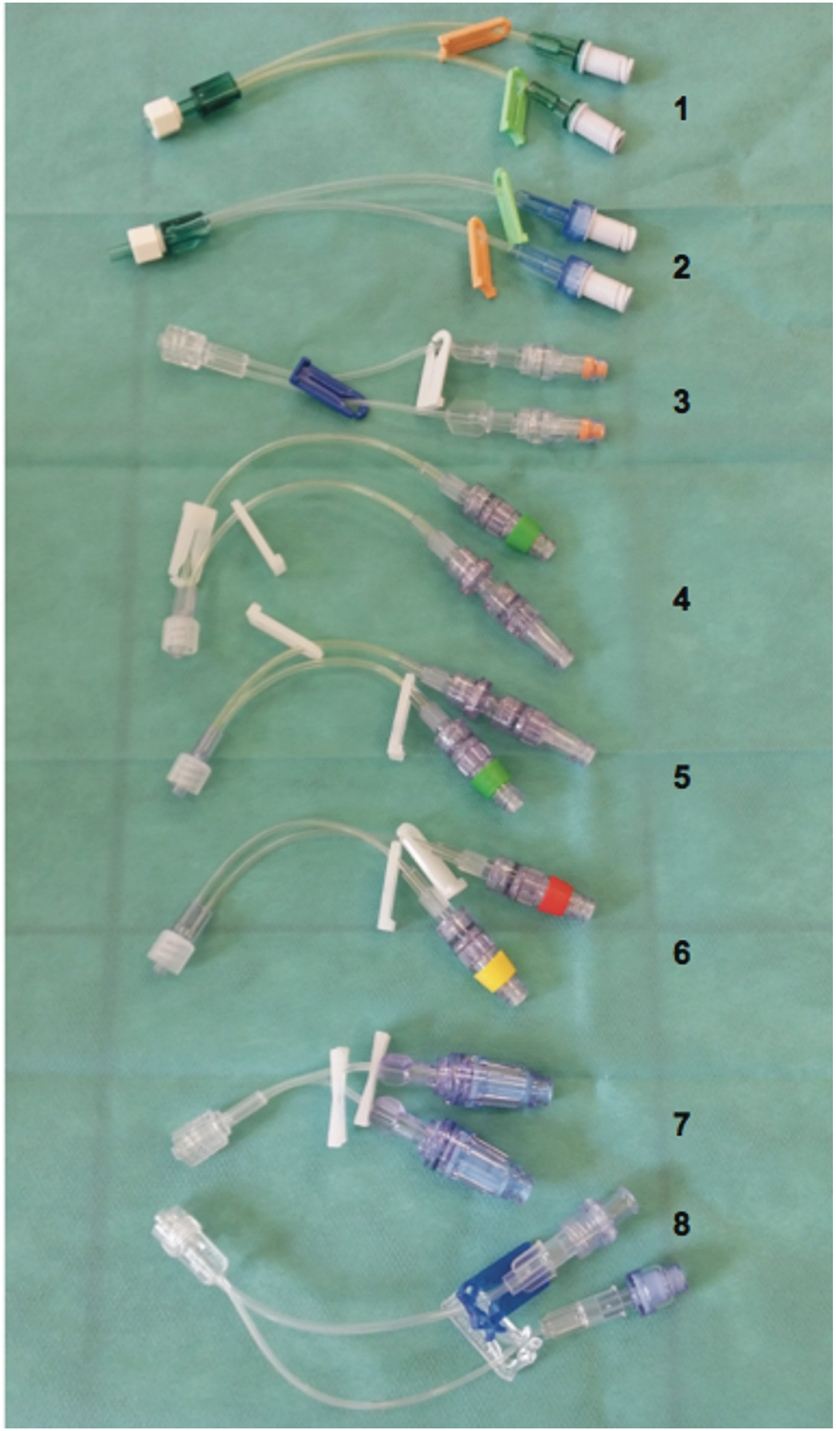
Double-lumen extension tubes made from different types of plastics (PUR, PVC and PE) assessed in our study. 1) Octopus 2 ref. 841.264. 2) Octopus 2 ref. 5841.208. 3) Spider ref. PY2101NCM. 4) Smallbore ref. 011-MC33076. 5) Smallbore ref. 011-MC33077. 6) Smallbore ref. 011-MC33165. 7) MaxPlus Clear ref. MFX2502MP. 8) Edelvaiss CW2+.

The study compared eight double-lumen extension tubes available on the French market in PUR, co-extruded (PE/PVC) plastic and plasticised PVC provided by different manufacturers (Fig 1 and Table 1).

**Table 1:** Characteristics of double-lumen extension tubes used for our study.

Double-lumen extension tube	Manufacturer	Reference	Type of plastic	Characteristics	Internal surface (mm^2^)	Type of sterilisation
Octopus 2	Vygon	841.264	PUR	Ø = 1.50 x 2.50 mm, L = 10 cm, Vol. = 0.34 mL	471	Gamma irradiation
Octopus 2	Vygon	5841.208	PUR	Ø = 1.50 x 2.50 mm, L = 10 cm, Vol. = 0.44 mL	471	Ethylene oxide gas
Spider double lumen	Cair LGL	PY2101NCM	PUR	Ø = 1 x 2 mm, L = 8 cm, Vol. = 0.40 mL	251.20	Ethylene oxide gas
Smallbore double lumen	ICU Medical	011-MC33076	PUR	Ø = 1.2 x 2.1 mm, L = 12 cm, Vol. = 0.51 mL	452.16	Gamma irradiation
Smallbore double lumen	ICU Medical	011-MC33077	PUR	Ø = 1.2 x 2.1 mm, L = 8 cm, Vol. = 0.47 mL	301.44	Gamma irradiation
Smallbore double lumen	ICU Medical	011-MC33165	PVC	Ø = 1.2 x 2.1 mm, L = 9 cm, Vol. = 0.43 mL	339.12	Gamma irradiation
MaxPlus Clear 2 way connector	Carefusion	MFX2502MP	PVC	Ø = 1.0 x 0.55 mm, L = 5 cm, Vol. > 0.50 mL	172.70	Ethylene oxide gas
Edelvaiss-CW2+	Doran International	Edelvaiss-CW2+	PE/PVC	Ø = 0.7 x 1.7 mm, L = 12 cm, Vol. = 0.05 mL	263.76	Ethylene oxide gas

PUR: polyurethane, PVC: polyvinyl chloride, PE: polyethylene

Ø: diameter, L: length, Vol.: volume (per tube).

### Impact of infusion device on drug delivery

Drug solutions were prepared from powder forms of active pharmaceutical ingredients: isosorbide dinitrate (Isosorbide Dinitrate CRS, European Pharmacopoeia Reference Standard, ref. I0775000, vial of 350 mg, Council of Europe—EDQM, Strasbourg, France) and diazepam (Diazepam, vial of 25 g, Cooper, Melun, France). Solutions of isosorbide dinitrate (200 μg/mL) and diazepam (7.5 μg/mL) were prepared by dissolving in an isotonic saline solution (0.9%NaCl, 500mL Viaflo^®^, Baxter, Maurepas, France) in a glass vial before using immediately. New syringes in polypropylene (BD Plastipak 20mL syringes) were prepared every day as reservoirs for the drug solutions and connected to extension lines in PE (Lectro-cath, réf. 1155.10, Vygon, L. 100 cm, Ø 1.0 x 2.0 mm, Vol. 1.1 mL) for which no sorption had been observed in a preliminary work (data not shown). Extension lines were purged with saline, using the automatic syringe pump function. According to clinical practices in adults, they were infused via syringe pumps (model Alaris^®^ CC, Carefusion, Voisins-le-Bretonneux, France) with a constant flow rate of 2.5 mL/h for isosorbide dinitrate and diazepam (n = 5 syringes per reference).

Drug concentrations in the prepared syringes and at the egress of extension tubes were determined over time by an analytical spectrophotometric UV-Vis method with a UV-Vis spectrophotometer (UV-2550 spectrophotometer, Shimadzu, Marne La Vallée, France) reading at λ = 220 nm for isosorbide dinitrate and 235 nm for diazepam to determine their sorption kinetics. The catheter egress was connected to the 10-mm UV spectrophotometer quartz cell (ref. 178.710-QS, Suprasil^®^, Hellma Analytics, Müllheim, Germany, V = 0.080 mL) through the inlet tube of the spectrophotometer flow cell (V = 0.137 mL) to measure drug concentrations continuously. Concentration values were recorded every minute over a total period of 150 minutes. All data was collected with UVProbe software ver. 2.31 (Shimadzu Corporation, Kyoto, Japan). Initial drug concentrations in syringes were measured with the spectrophotometric UV_Vis method which was validated with six concentrations of between 25 and 400 μg/mL for isosorbide dinitrate and seven concentrations of between 1 and 15 μg/mL for diazepam, repeated six times by the same person. The limits of detection (LOD) and quantification (LOQ) were respectively 2.78 and 5.57 μg/mL for isosorbide dinitrate and 0.11 and 0.21 μg/mL for diazepam.

To identify factors other than sorption, control experiments were performed using norepinephrine (Noradrenaline tartrate (2 mg/mL), Aguettant, Lyon, France), which is known not to absorb to plastics. Norepinephrin was prepared with noradrenaline tartrate in saline for drug concentration at 100 μg/mL and infused in the same conditions as isosorbide dinitrate and diazepam at a constant flow rate of 2.5 mL/h (n = 3 syringes per reference).

Drug concentrations (expressed in percentage of concentration values measured in syringes prepared at T0) from the different double-lumen extension tubes were measured at T0+150 min. As the variation in drug delivery at the beginning of infusion depends on the internal volume of the tube, we did not take into account the first measurements which correspond to the rapid increase in drug concentration delivered at the egress of the extension tube. Values were compared statistically, using analysis of variance (ANOVA). When this revealed a significant p value (p<0.05), contrasts were established with the Tukey test to detect significant differences between pairs of infusion devices. Results are expressed as mean values ± standard deviation in percentage. Mean concentrations of sorbed drug ± standard deviation were also determined for each tube at T0+150 min, as follows: [drug concentration measured at T0+150 min / drug concentration measured in syringe at T0] / inner surface of the cylinder. Results are expressed in % per mm^2^.

### Assessment of plastic material properties for PUR infusion devices

For PUR, the main characteristics of the plastics were determined through different tests [21]:

- Elastic properties under the effect of heat. For each double-lumen extension tube in PUR, a tube length of 10 cm was sampled and placed on a hot plate heated to over 200°C and then stretched manually to determine whether the plastic was deformable or not.
- Resistance properties to stretching. A tube length of 10 cm was immersed in 50 mL of methanol (Technical grade, VWR Chemicals) at room temperature. After 24 hours’ immersion, it was stretched manually to assess its resistance properties to breakage.

## Results

### Impact of infusion device on drug delivery

**Fig 2:**
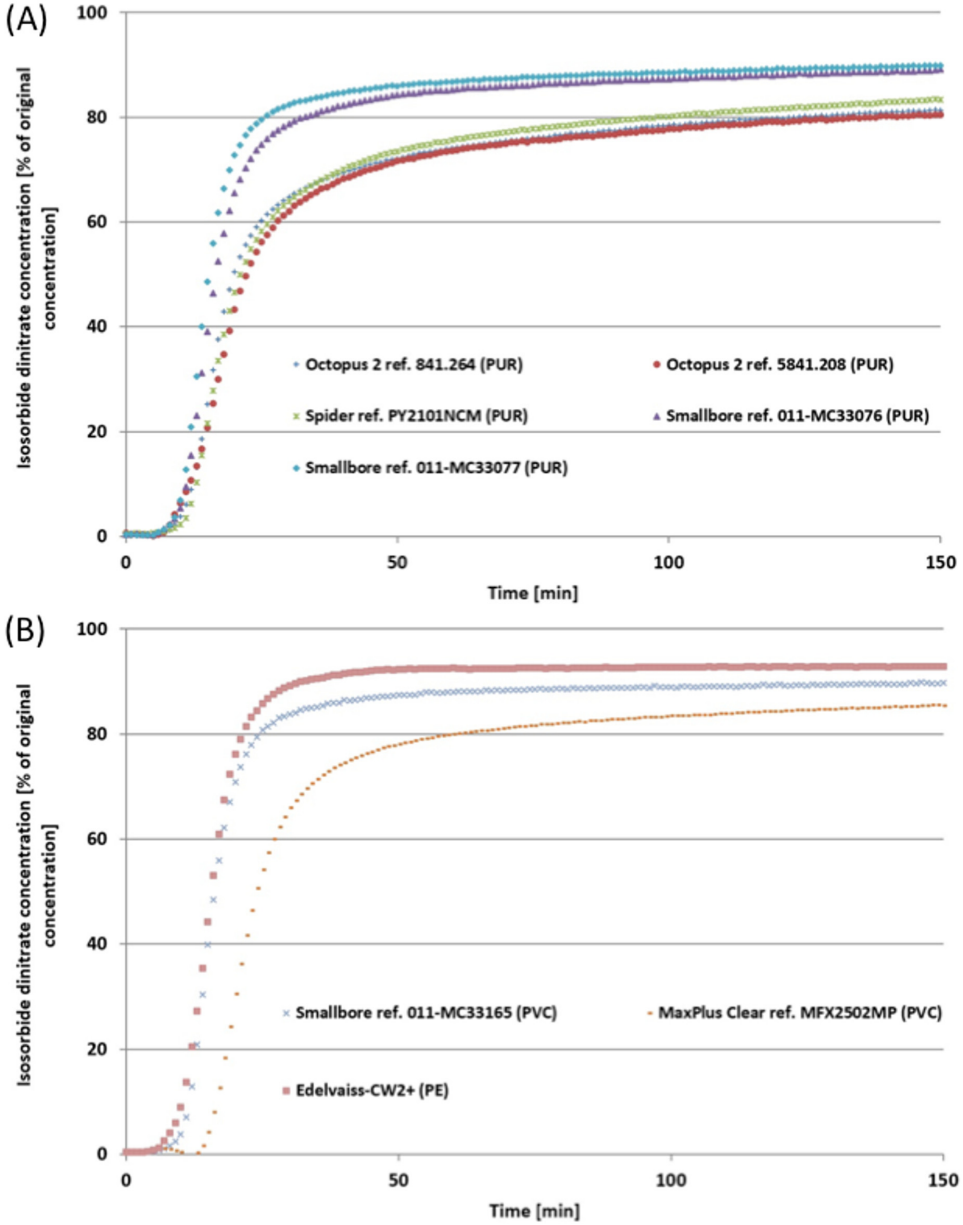
Mean concentrations of isosorbide dinitrate (as % of the initial syringe concentrations) delivered at the egress of the double-lumen extension tubes during the 150-minute simulated infusion. (A) PUR tubes. (B) PVC and PE tubes.

**Fig 3:**
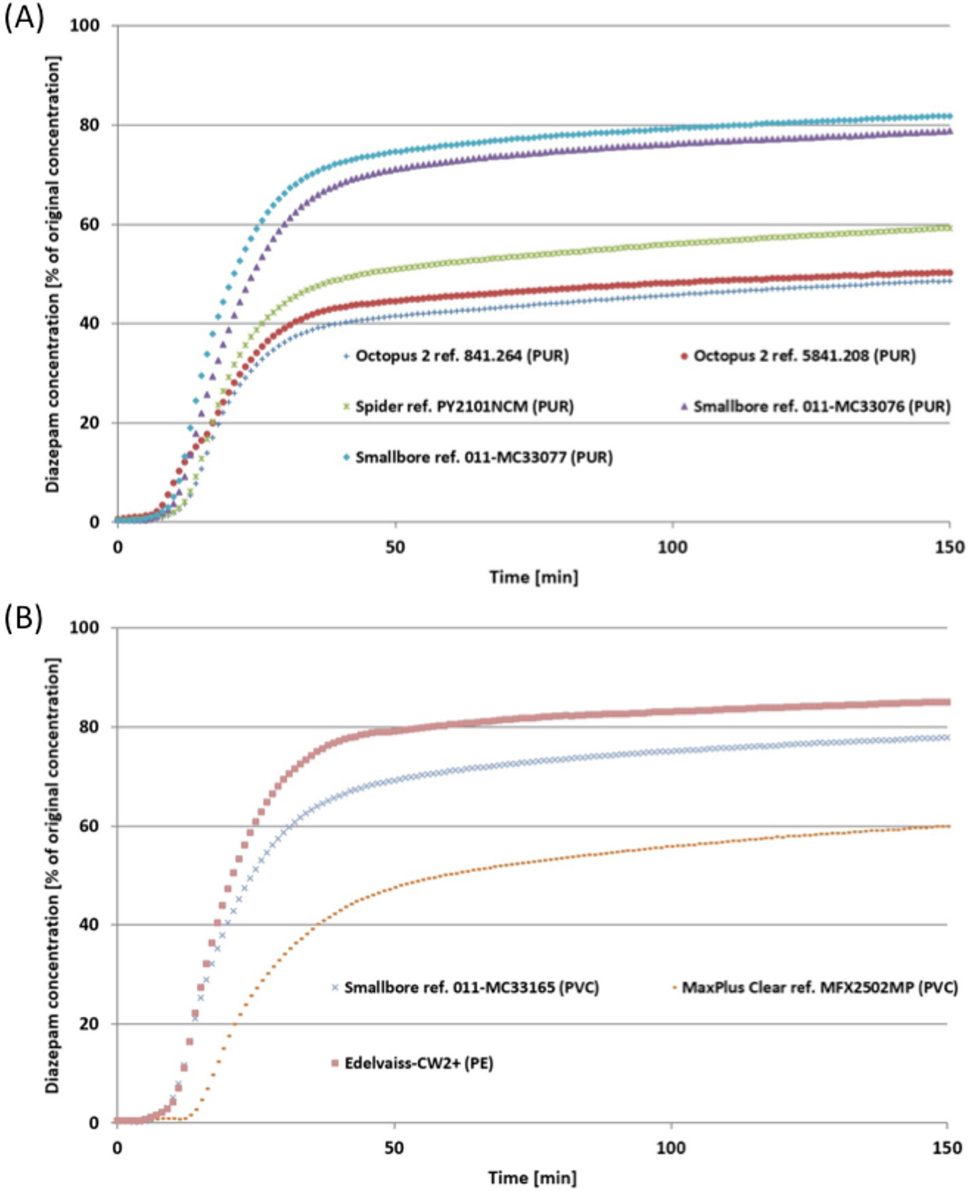
Mean concentrations of diazepam (as % of the initial syringe concentrations) delivered at the egress of the double-lumen extension tubes during the 150-minute simulated infusion. (A) PUR tubes. (B) PVC and PE tubes.

**Fig 4:**
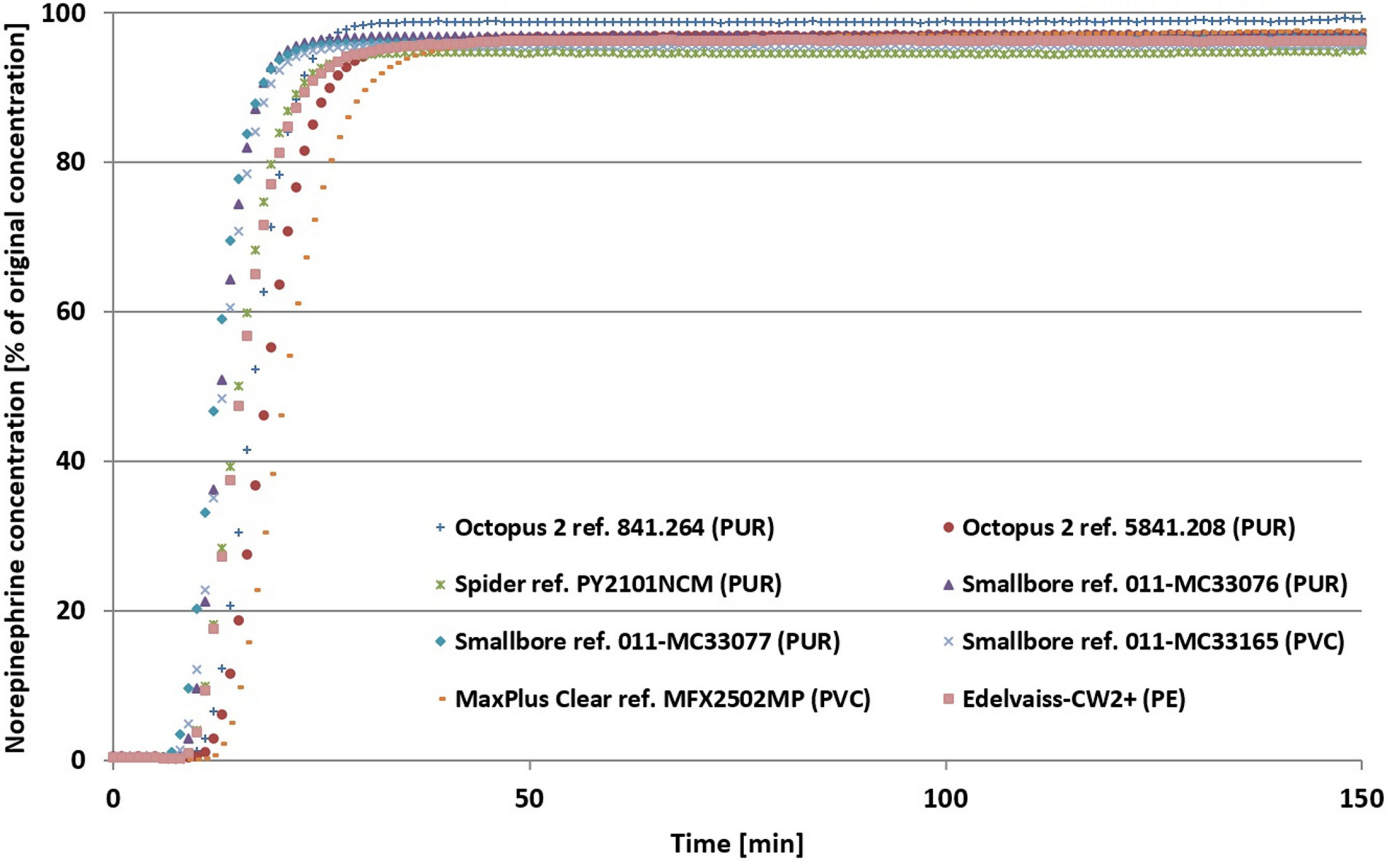
Mean concentrations of noradrenaline (as % of the initial syringe concentrations) delivered at the egress of the double-lumen extension tubes during the 150-minute simulated infusion.

Figs 2, 3 and 4 show mean concentrations of diazepam, isosorbide dinitrate and norepinephrine at the egress of double-lumen extension tubes made of different materials (PUR, PVC and PE) during a simulated infusion lasting 150 minutes. Generally, the curves revealed that drug concentrations vary during infusion. In fact, after 10 minutes’ infusion, the drug concentration profile was different from one tube to another. Norepinephrine curves revealed the impact of extension set design during this first phase of infusion. After 40 minutes, drug sorption was at a maximum and at the end of infusion, the concentration of the drug was still significantly lower than the initial concentration in the syringe, whatever the plastic. The first finding was therefore that the nature of the drug, and particularly the choice of material, impact on drug concentration at the tube egress.

The double-lumen extension tube in PE (Edelvaiss-CW2+) had the lowest drug sorption with an egress concentration of 85.06% and 92.84% of the initial concentration after a 150-minute infusion for diazepam and isosorbide dinitrate, respectively (Figs 2B and 3B). Double-lumen extension tubes in PVC show different results, according to the manufacturer. On the one hand, the Smallbore tube ref. 011-MC33165 (ICU Medical) had low adsorption comparable to that observed with the PE tube (Edelvaiss-CW2+) after the same infusion period (egress concentrations: 77.89% vs. 85.06% for diazepam; 89.75% vs. 92.84% for isosorbide dinitrate). On the other hand, the MaxPlusClear tube ref. MFX2502MP (Carefusion) revealed high adsorption with diazepam and isosorbide dinitrate concentrations of 59.99% and 85.43% respectively at the egress of the tubes during the same infusion period (Figs 2B and 3B).

Double-lumen extension tubes in PUR also showed different drug delivery and sorption profiles during the 150-minute infusion of drug solutions. Manufacturers concord that in terms of concentration, results at the egress of tubes are different and two groups are clearly distinguishable. A first group comprises two references of Smallbore tubes (011-MC33076 and 011-MC33077) showing low adsorption similar to that observed on the PE tube (Edelvaiss CW2+) with both diazepam (78.83%—81.88%) and isosorbide dinitrate (89.15–89.81%). A second group consists of two references of Octopus tubes (841.264 and 5841.208) and the Spider tube which show a strong and similar adsorption to that observed on the MaxPlus Clear tube in PVC with both diazepam (48,58%—50.30%—59.30%) and isosorbide dinitrate (81.37%—80.53%—83.40%) (Tables 2 and 3).

**Table 2:** Mean concentrations of isosorbide dinitrate (as % of the initial syringe concentrations) delivered at the egress of the double-lumen extension tubes and mean concentrations of isosorbide dinitrate sorbed per internal surface (in % per mm^2^) at T0+150 min.

Double-lumen extension tube	Reference	Tubing material	Mean concentrations of isosorbide dinitrate delivered (%)	Mean concentrations of isosorbide dinitrate sorbed per internal surface (% per mm^2^)
Octopus 2	841.264	PUR	81.37 ± 2.35	0.040 ± 0.005^1^
Octopus 2	5841.208	PUR	80.53 ± 1.66	0.041 ± 0.004^2^
Spider double lumen	PY2101NCM	PUR	83.40 ± 0.61	0.066 ± 0.002^3^
Smallbore double lumen	011-MC33076	PUR	89.15 ± 2.09	0.024 ± 0.005^4^
Smallbore double lumen	011-MC33077	PUR	89.81 ± 1.40	0.034 ± 0.005^5^
Smallbore double lumen	011-MC33165	PVC	89.75 ± 1.16	0.030 ± 0.003^6^
MaxPlus Clear 2 way connector	MFX2502MP	PVC	85.43 ± 1.22	0.084 ± 0.007^7^
Edelvaiss-CW2+	Edelvaiss-CW2+	PE/PVC	92.84 ± 2.73	0.027 ± 0.010^8^

PUR: polyurethane, PVC: polyvinyl chloride, PE: polyethylene

Mean concentrations of isosorbide dinitrate sorbed per internal surface at T0+150 min are significantly different (analysis of variance: ANOVA, p ≤ 0.0001).

^1^ Significant difference vs. Spider, Smallbore 011-MC33076, MaxPlus Clear and Edelvaiss-CW2+ (Tukey test, p ≤ 0.030)

^2^ Significant difference vs. Spider, Smallbore 011-MC33076, MaxPlus Clear and Edelvaiss-CW2+ (Tukey test, p ≤ 0.009)

^3^ Significant difference vs. Octopus 2 ref. 841.264, Octopus 2 ref. 5841.208, Smallbore 011-MC33076, Smallbore 011-MC33077, Smallbore 011-MC33165, MaxPlus Clear and Edelvaiss-CW2+ (Tukey test, p < 0.0001)

^4^ Significant difference vs. Octopus 2 ref. 841.264, Octopus 2 ref. 5841.208, Spider and MaxPlus Clear (Tukey test, p ≤ 0.001)

^5^ Significant difference vs. Spider and MaxPlus Clear (Tukey test, p < 0.0001)

^6^ Significant difference vs. Spider and MaxPlus Clear (Tukey test, p < 0.0001)

^7^ Significant difference vs. Octopus 2 ref. 841.264, Octopus 2 ref. 5841.208, Spider, Smallbore 011-MC33076, Smallbore 011-MC33077, Smallbore 011-MC33165 and Edelvaiss CW2+ (Tukey test, p < 0.0001)

^8^ Significant difference vs. Octopus 2 ref. 841.264, Octopus 2 ref. 5841.208, Spider and MaxPlus Clear (Tukey test, p ≤ 0.030).

**Table 3:** Mean concentrations of diazepam (as % of the initial syringe concentrations) delivered at the egress of the double-lumen extension tubes and mean concentrations of diazepam sorbed per internal surface (in % per mm^2^) at T0+150 min.

Double-lumen extension tube	Reference	Tubing material	Mean concentrations of diazepam delivered (%)	Mean concentrations of diazepam sorbed per internal surface (% per mm^2^)
Octopus 2	841.264	PUR	48.58 ± 2.88	0.109 ± 0.006^1^
Octopus 2	5841.208	PUR	50.30 ± 1.46	0.106 ± 0.003^2^
Spider double lumen	PY2101NCM	PUR	59.30 ± 1.21	0.162 ± 0.005^3^
Smallbore double lumen	011-MC33076	PUR	78.83 ± 2.26	0.047± 0.005^4^
Smallbore double lumen	011-MC33077	PUR	81.88 ± 2.49	0.060 ± 0.008^5^
Smallbore double lumen	011-MC33165	PVC	77.89 ± 2.51	0.065 ± 0.007^6^
MaxPlus Clear 2 way connector	MFX2502MP	PVC	59.99 ± 1.24	0.232 ± 0.007^7^
Edelvaiss-CW2+	Edelvaiss-CW2+	PE/PVC	85.06 ± 3.94	0.057 ± 0.015^8^

PUR: polyurethane, PVC: polyvinyl chloride, PE: polyethylene

Mean concentrations of diazepam sorbed per internal surface at T0+150 min are significantly different (analysis of variance: ANOVA, p <0.0001).

^1^ Significant difference vs. Spider, Smallbore 011-MC33076, Smallbore011-MC33077, Smallbore011-MC33165, MaxPlus Clear and Edelvaiss-CW2+ (Tukey test, p < 0.0001)

^2^ Significant difference vs. Spider, Smallbore 011-MC33076, Smallbore011-MC33077, Smallbore011-MC33165, MaxPlus Clear and Edelvaiss-CW2+ (Tukey test, p < 0.0001)

^3^ Significant difference vs. Octopus 2 ref. 841.264, Octopus 2 ref. 5841.208, Smallbore 011-MC33076, Smallbore011-MC33077, Smallbore011-MC33165, MaxPlus Clear and Edelvaiss-CW2+ (Tukey test, p < 0.0001)

^4^ Significant difference vs. Octopus 2 ref. 841.264, Octopus 2 ref. 5841.208, Spider, Smallbore 011-MC33165 and MaxPlus Clear (Tukey test, p ≤ 0.015)

^5^ Significant difference vs. Octopus 2 ref. 841.264, Octopus 2 ref. 5841.208, Spider and MaxPlus Clear (Tukey test, p < 0.0001)

^6^ Significant difference vs. Octopus 2 ref. 841.264, Octopus 2 ref. 5841.208, Spider, Smallbore 011-MC33076 and MaxPlus Clear (Tukey test, p ≤ 0.015)

^7^ Significant difference vs. Octopus 2 ref. 841.264, Octopus 2 ref. 5841.208, Spider, Smallbore 011-MC33076, Smallbore 011-MC33077, Smallbore011-MC33165 and Edelvaiss CW2+ (Tukey test, p < 0.0001)

^8^ Significant difference vs. Octopus 2 ref. 841.264, Octopus 2 ref. 5841.208, Spider and MaxPlus Clear (Tukey test, p < 0.0001).

**Fig 5:**
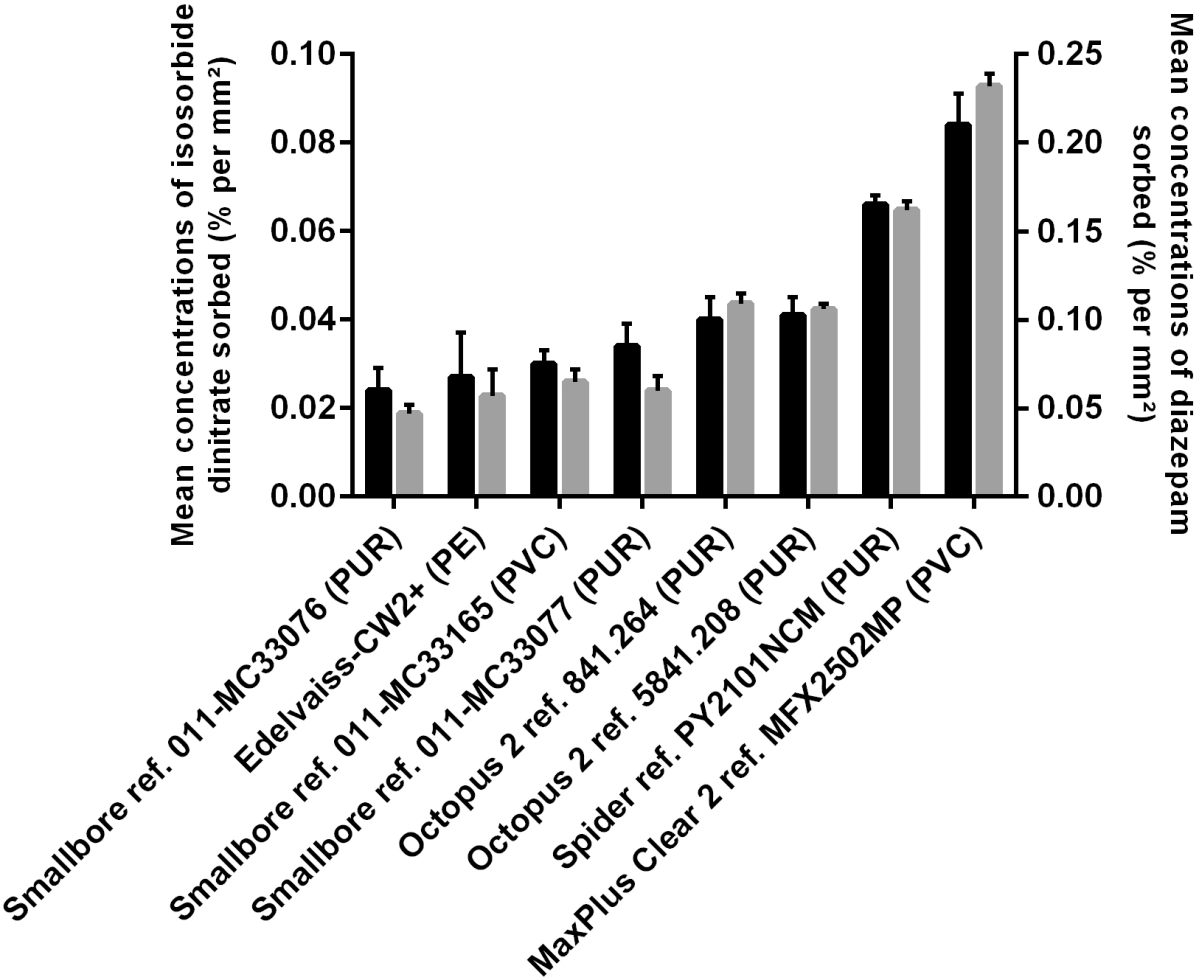
Mean concentrations of drug sorbed per internal surface (in % per mm^2^) at T0+150 min for each double-lumen extension tube investigated in the case of isosorbide dinitrate and diazepam.

Fig 5 presents the mean concentrations of drug sorption per internal surface (in % per mm^2^) when the steady state was reached so as to normalise the results. In general, the same classification of sorption profiles was found, whatever the drug (Tables 2 and 3). As expected, the reference Edelvaiss-CW2+ (PE) is one of those which sorbs the least (0.027 and 0.057% per mm^2^ for isosorbide dinitrate and diazepam, respectively), MaxPlusClear ref. MFX2502MP (PVC) is the one which sorbs the most, 4 times more than PE (0.084 and 0.232% per mm^2^ for isosorbide dinitrate and diazepam, respectively). The Smallbore references (PUR and PVC) sorb a similar amount of drug to PE. The Octopus and Spider references (PUR) sorb twice as much as PE regardless of the drug. We propose a classification of commercial tubes using these two drugs from the least sorbent to the most sorbent: Edelvaiss CW2+ (PE) > Smallbore (PUR, PVC) > Octopus (PUR) > Spider (PUR) > MaxPlusClear (PVC).

The second finding was that, for the same material, there are different compositions (plasticisers) or different degrees of cross-linking, which impact the adsorption properties of the tube.

### Assessment of the plastic properties of PUR infusion devices

Further study of the different PUR tubes was conducted to determine their thermoplastic/thermosetting behaviour. Table 4 shows their different properties. Characteristics of plastics vary from manufacturer to manufacturer. Combinations of elastic and resistance properties are different for the eight tube references, which means that PUR can be either thermoplastic or thermosetting. Indeed, even if the chemical composition is identical, the degree of polymerisation makes PUR thermoplastic or thermosetting. Table 2 shows that all the PUR tubes did not have the same behaviour after immersion in a solvent or after heating. The Octopus and Spider tubes had thermoplastic behaviour whereas the Smallbore tube had thermosetting behaviour (like resin). The third finding was that the degree of crosslinking (thermoplastic or thermosetting) of the same polymer (PUR) has an impact on the adsorption properties of the tubes.

**Table 4:** Determining the type of plastic (thermoplastic or thermosetting) for PUR double-lumen extension tubes through different tests (heat resistance and reaction in an organic solvent-type methanol).

Double-lumen extension tube	Manufacturer	Reference	Heat resistance	Reaction in an organic solvent (methanol)	Type of PUR
Octopus 2	Vygon	841.264	Deformable	Stretching	Thermoplastic
Octopus 2	Vygon	5841.208	Deformable	Stretching	Thermoplastic
Spider double lumen	Cair LGL	PY2101NCM	Deformable	Breaking	Thermoplastic
Smallbore double lumen	ICU Medical	011-MC33076	Non-deformable	Stretching	Thermosetting
Smallbore double lumen	ICU Medical	011-MC33077	Non-deformable	Stretching	Thermosetting

PUR: polyurethane.

The two PVC tubes were no different in terms of elongation after heating or immersion in a solvent; both were thermoplastic. The plasticisers used were the same (TOTM) and cannot account for the difference in drug adsorption. The difference is in the sterilisation process. Indeed, Table 1 shows that reference ICU was sterilised with gamma radiation and reference Carefusion by ETO. PVC is sensitive to gamma radiation, which can cause changes in its chemical structure leading to increased PVC crosslinking and decreased drug adsorption. [22,23]

The fourth finding was that the sterilisation process has an impact on the adsorption properties of the tubes.

## Discussion

The results of this study reveal that 1) small double-lumen extension tubes may interact significantly with infused drugs and 2) this interaction depends on tube material, the drug, cross linking and sterilisation. Even if the contact time between solution and tube material is low, drug sorption into the tube material is significant (up to 51% of the original concentration for diazepam and up to 19% for isosorbide dinitrate). The type of tube material (PUR, PVC and PE) as well as the type of sterilisation process is a factor influencing drug sorption behaviour. The amount of sorption decreases over time and the concentration of the active pharmaceutical compound in the solution during infusion reaches a steady state at the end of the application time. Nevertheless, the concentration at the egress of the catheter remains below the expected concentration. These results confirm that it is difficult to administer a predictable concentration of drug to a patient, especially for low-concentrated drug solutions. [24,25] Other alternative tube materials assessed, such as PUR or PE, showed significantly lower sorption than PVC tubes. However, some references in PUR such as Octopus 2 ref. 841.264 and 5841.208 and Spider ref. PY2101NC had significantly higher sorption than PVC tubes.

Isosorbide dinitrate and diazepam were chosen for this study as model compounds for sorption as previous references in research literature have indicated that they may exhibit significant sorption into plastics like PVC and possible alternatives. [26,27] The amount of sorption is related to the physicochemical properties of the polymer, the drug compounds and the contact media in which the drug is dissolved. Isosorbide dinitrate and diazepam were dissolved in 0.9% sodium chloride solutions in a glass vial before use. However, the two active pharmaceutical ingredients were not sorbed in the same way, whatever the tube studied. Isosorbide dinitrate showed a lower sorption profile than diazepam. The amount of sorption depends on drug interaction with the plasticised surface of an infusion system. [28] The diffusion of diazepam into the polymer matrix appeared to be higher than that of isosorbide dinitrate, resulting in more significant sorption behaviour during infusion through the investigated tubes. Deeper insight is required into the interaction of different drug solutions/application set combinations to be able to evaluate the concentration throughout flow time of the active pharmaceutical ingredient over administration time to the patient.

The study shows that drug concentrations are significantly different, firstly for all the tubes investigated, then when compared in pairs, and even for tubes in the same plastic material. Contrary to what had been expected, the PUR tubes showed the greatest loss of drug at the end of the application time. Isosorbide dinitrate behaved similarly to diazepam. The sorption behaviour of PUR tubings was significantly different when the material was either thermosetting or thermoplastic.

Experiments with norepinephrine highlighted the impact of extension set design during the first stage of infusion with saline-filled tubes prior to the onset of drug infusion. These experiments illustrated the impact of dead volume on the delay to reach the steady state. [4]

Several approaches have already been published to determine the sorption behaviour of drugs in tubes. For each drug solution and target concentration, it is difficult to predict drug sorption on/in a plastic material for an individual administration set because of the nature of the plastic and the associated sterilisation process.

However, there are some limitations to our study. The results should be confirmed with other formulations/concentrations, not only with isosorbide dinitrate and diazepam but also with other drugs, as sorption of the active ingredient into the administration set may well be affected. Other infusion flow rates should be tested, as should the total duration of the application which determines the contact time between drug solution and plastic material. Assessment should be made of other plastic tube materials and of the possibility of stabilising drug concentration by pre-conditioning the tube with an infusion of the concentrated drug solution. These parameters may influence the concentration profile of the drug solution in the tube at equilibrium. Moreover, our method does not enable us to assess the adsorption kinetics of double-lumen extension tubes because the extension tubes are previously purged with a saline volume relative to the internal volume of tubes, making the first phase of the curves impossible to interpret.

## Conclusions

The results of this study reveal firstly, that small double-lumen extension tubes may interact significantly with infused drugs and secondly, this interaction depends on tube material, the drug itself, cross linking of the plastic (PUR) and perhaps the sterilisation process. Clinicians must be aware of potential drug interactions with the materials of extension tubes even if of short length and must consider the nature of tube material when choosing their infusion devices.
